# Distribution of Peptidyl-Prolyl Isomerase (PPIase) in the Archaea

**DOI:** 10.3389/fmicb.2021.751049

**Published:** 2021-10-07

**Authors:** Anchal, Vineeta Kaushik, Manisha Goel

**Affiliations:** Department of Biophysics, University of Delhi South Campus, New Delhi, India

**Keywords:** archaea, PPIase, structural analysis, distribution, *cis*-*trans* isomerization

## Abstract

Cis-trans isomerization of the peptide bond prior to proline is an intrinsically slow process but plays an essential role in protein folding. *In vivo cis-trans* isomerization reaction is catalyzed by Peptidyl-prolyl isomerase (PPIases), a category of proteins widely distributed among all the three domains of life. The present study is majorly focused on the distribution of different types of PPIases in the archaeal domain. All the three hitherto known families of PPIases (namely FKBP, Cyclophilin and parvulin) were studied to identify the evolutionary conservation across the phylum archaea. The basic function of cyclophilin, FKBP and parvulin has been conserved whereas the sequence alignment suggested variations in each clade. The conserved residues within the predicted motif of each family are unique. The available protein structures of different PPIase across various domains were aligned to ascertain the structural variation in the catalytic site. The structural alignment of native PPIase proteins among various groups suggested that the apo-protein may have variable conformations but when bound to their specific inhibitors, they attain similar active site configuration. This is the first study of its kind which explores the distribution of archaeal PPIases, along with detailed structural and functional analysis of each type of PPIase found in archaea.

## Introduction

Protein folding is the process by which the linear information contained in the polypeptide sequence gives rise to the well-defined three-dimensional conformation of the functional protein (Hartl, 1996). This process must be achieved rapidly and effectively to avoid aggregation and misfolding, as the folding intermediates have the tendency to interact with one another (Netzer and Hartl, 1998) and unwarranted interactions not only preclude proper protein folding but may also lead to aggregation making the cell environment toxic. Thereby, molecular chaperones and foldases play an essential role in expediting the protein folding process (Hartl and Hayer-Hartl, 2002; Zhang et al., 2013). The foldases type of molecular chaperones include protein disulfide isomerases, which catalyze formation and isomerization of disulfide bonds and peptidyl-prolyl *cis-trans* isomerases (PPIases), which catalyze the *cis-trans* isomerization of peptide bonds in protein folding (Brandts et al., 1975; Schmid, 1993). The peptide bond linking adjacent amino acid residues in a protein backbone can adopt either *trans* or *cis* conformation. Proline is unique among all biological amino acids which has the ability/tendency to adapt completely distinct *cis* and *trans* conformation. Many Biological process has time scale of milliseconds, while *cis- trans* isomerization of peptidyl-prolyl imide bond is extremely slow in its dynamics, hence a rate-limiting step in protein Folding (Velazquez and Hamelberg, 2015). PPIases have been shown to help overcome this limitation in the protein folding process by decreasing the energy barrier between *cis* and *trans* conformation (Ou et al., 2001).

It has been over 100 years since the discovery of first reported PPIases in 1989, when porcine kidney PPIase was purified and shown to be similar to bovine cyclophilin, a target for immunosuppressant, cyclosporine A (Fischer et al., 1989). After this discovery, FK506 which is an immunosuppressant, was isolated from *Streptomyces tsukubaensis* no. 9993 (Kino et al., 1987) and protein exhibiting binding to FK506, termed as FKBP was discovered and shown to have PPIase activity (Harding et al., 1989). In 1994, the third family of PPIases, Parvulin was discovered in *Escherichia coli.* Hence, PPIases consists of proteins that are categorized into three families. These three families exhibit no sequence similarity to one other but FKBP and PPIase domain of parvulin resembles with each other in structure, however cyclophilins appear to be different (Suzuki et al., 2003). Even after a century of PPIase discovery, most of the study exploring their function and role in protein folding has been carried out in only eukaryotes, and while several reports are available for bacteria, very few studies have aimed at archaeal organisms.

Peptidyl-prolyl isomerase are ubiquitous in all three domains: eukaryotes, bacteria and archaea. It was observed that number of paralogs of PPIases increased from bacteria to human as *E. coli* has two cyclophilins, four FKBPs, a trigger factor, and two parvulins (Pahl et al., 1997),the budding yeast *Saccharomyces cerevisiae* has seven cyclophilins, four FKBPs, and a parvulin, and human genome codes for 18 different cyclophilins and many variants of FKBP and parvulin. Various cyclophilin and FKBPs homologs are known from the past many years but the distinct function of each is not yet clear. Both, the cyclophilin and FKBP, are mainly involved in folding of newly synthesized protein gathering and transporting the cellular protein complexes (Kang et al., 2008). Pin1 (type of parvulin) has been reported to play a significant role in the cell cycle mechanism of eukaryotes (Lin et al., 2015).

Most of the proteins undergo structural denaturation under harsh environmental conditions such as heat shock, change in pH etc. all collectively termed as cell stress. However, there is specific set of organisms that can tolerate these conditions quite well and are therefore categorized as extremophiles. Archaea thrive in many extremes like heat, cold, acid, base, salinity pressure and radiations. Archaea have evolved over time adapt and thrive in such environments by only having few protein modifications, which makes its enzyme active over a range of conditions (Reed et al., 2013). Archaeal proteins show several adaptations such as increased number of large hydrophobic residues, disulfide bonds and ionic interactions that give these proteins the advantage of thermal stability at extremes of temperature (Reed et al., 2013). However, not all adaptations can be explained only through these changes in the amino acid sequence. It is possible that archaea have some adaptations in their protein folding and proteostasis machinery which helps these organisms maintain viability at conditions unsuitable to other organisms, PPIases are one class of proteins which need to be studied in archaea as, these proteins are reported to perform various important function of protein folding in eukaryotes and bacteria.

Peptidyl-prolyl isomerase are known to be involved in protein folding *via* their PPIase activity but have also been shown to have chaperone like activity, so it is hypothesized that these proteins can be stress related protein which may be helpful in fastening the protein folding step and maturation of newly synthesized protein under cell stress condition. Expression of different cyclophilins and FKBPs gene have been shown to be enhanced in response to different abiotic stress conditions. Cyclophilin gene OsCYP-25 (LOC_Os09 g39780) from rice was found to be upregulated in response to various abiotic stresses viz., salinity, cold, heat and drought. FKBP12 gene is induced in *Polytrichastrum alpinum* by heat ABA, drought and salt stress. Overexpression of PaFKBP12 in Arabidopsis increased the plant size, which induced the cell cycle and showed a better growth and survival as compared to wild type variety. Apart from role of PPIase in stress conditions of plants they also help in regulation of other cell process in various organisms. *C. elegans* is normally grown at temperatures between 16°C and 25°C. When the worms are exposed to a low temperature (10°C), the expression level of parvulin increases, the increased expression of Pin-4 indicates a possible role in the worm’s adaptation at lower temperature. Hence, PPIases are expected to play important role in and organisms adaptation to extreme conditions.

Very little information, however, is available for archaeal PPIases. In archaea, *Halobacterium cutirubrum* cyclophilin was the only functionally characterized cyclophilin until recently. The biophysical and biochemical characterization of cyclophilin protein from archaeal organism *Methanobrevibacter ruminantium* was recently reported (Kaushik et al., 2019). Two types of FKBPs, long-type (26–30 kDa) and short-type FKBP (17–18 kDa), have been found in archaea which is a unique characteristic of archaeal organism. Although a few of the long-type and short-type FKBPs have been characterized (Maruyama et al., 2004), the difference in properties and the need to have two different types of FKBPs in these organisms is not understood yet. Two structural studies of Parvulin are known till now from archaeal organisms *Cenarchaeum symbiosum and Nitrosopumilus maritimus* (Jaremko et al., 2011; Hoppstock et al., 2016). Archaea acts as evolutionary connecting link between eukaryotes and prokaryotes and based on comparative genomic studies it was found that molecular machinery of archaea resembles that found in eukaryotes (Koonin, 2015). In the present study, an attempt is made to summarize the distribution of PPIases in archaeal organisms along with copy numbers, predicted conserved motifs and structural variation among different groups in their native and inhibitor bound form. In this study extensive *in silico* analysis is done to gain new insights of archaeal PPIases.

## Materials and Methods

### Data Collection

The Primary objective was collection of PPIase homologs from completely annotated archaeal genomes. Completely sequenced 196 archaeal genomes were retrieved from NCBI using the limits: sequence location on chromosome and organisms group archaea. For analysis of PPIase protein sequences, proteome of completely sequenced archaeal genomes were retrieved from NCBI. A Standalone database was generated using the proteome information of 196 archaeal genomes.

### Construction of Proteome Database

To study the distribution of PPIases proteins in different archaeal classes and orders, a database of each family having proteome of each PPIase family was separately constructed. To extract the proteomic information of each family from these 196 archaeal genomes representatives, a protein seed sequence from each PPIase family was chosen. Standalone BLAST was run against the developed proteome database using *Picrophilus torridus* as a seed sequence to extract the proteome information of cyclophilin protein. As FKBPs are of two types: long-type and short-type FKBPs, two different seed sequences were used. long-type FKBP sequence from *Methanococcus jannaschii* (PDBID: 3PRB) and short-type FKBP from *Methanococcus jannaschii* (PDBID: 3PR9) was used as a seed sequence (Martinez-Hackert and Hendrickson, 2011). To extract parvulin homologs, *Cenarchaeum symbiosum* (PDBID: 2RQS) was used as a seed sequence (Jaremko et al., 2011). This generated database served as foundation to study the distribution of three types of PPIases proteins in Archaeal domain.

### Sequence Alignment and Phylogenetic Tree Construction of Peptidyl-Prolyl Isomerase Class

The multiple sequence alignment for each family of PPIase was generated using Clustal Omega (Madeira et al., 2019), GUIDANCE server (Penn et al., 2010). Clustal Omega is a multiple sequence alignment program that used seeded guide tree and HMM profile-profile techniques to generate alignment. GUIDANCE server uses three different alignment protocols: MAFT, ClustalW and PRANK. T-COFFEE was also used for generation of multiple sequence alignment and to access the score of generated multiple sequence alignment (Notredame et al., 2000). Incomplete and highly divergent sequences were omitted from the analysis. The final data set includes 106 cyclophilin homologs, 327 FKBP proteins and 30 parvulin sequences.

To study the evolution of each PPIase family, a phylogenetic tree was constructed using the MEGA 6.0 (Molecular Evolutionary Genetic Analysis) (Tamura et al., 2013). MEGA 6.0 is an integrated tool for making phylogenetic tree, inferring ancestral sequences and testing evolutionary hypothesis. The phylogenetic tree of the 106 archaeal cyclophilins, 327 FKBPs and 30 parvulin were constructed by Maximum Likelihood (ML) methods based on gamma corrected Jones-Tylor thorton using MEGA 6.0. The topology of constructed tree was evaluated through bootstrapping analysis based on 1,000 replicates.

### Motif Analysis by Using Multiple Expectation-Maximization for Motif Elicitation

To predict the conservation of amino acid residues in each PPIase family, which may be functionally important MEME (Multiple Expectation-maximization for Motif Elicitation) server was used (Bailey et al., 2009). This server is based on exception maximization algorithm. The motif prediction was performed by MEME server using OOPS model (at least 1 motif per sequence), and the length of motif was expected to be between 5 and 15 amino acids keeping other parameters as default. MEME builds a position-specific scoring matrix wherein there is a probability with the occurrence of each base at each position. Hence, information revealed in terms of motifs can be used to predict the conservation and functionally important amino acid residues in family (Wong et al., 2015).

### Structure Comparison Methodology

All the PDB structures and fasta sequences available for cyclophilin, FKBP and parvulin were retrieved from RCSB PDB. There is high redundancy in retrieved PDB entries so to filter out the data; CD-hit at 100% sequence identity was performed. After CD-hit, phylogenetic tree was constructed using MEGA 6.0 to analyze how native and substrate/inhibitor bound structures relate to each other in every group. From the phylogenetic tree, cluster which has native and substrate/inhibitor bound structure from same group were selected for the study as they could help in better decipherment of changes in native vs substrate or inhibitor bound structures.

## Results

There is no significant report till now about the distribution of archaeal PPIases in the archaeal kingdom. In eukaryotes and bacterial organisms, multiple copies of cyclophilin are present but no such information is available for archaeal cyclophilin. In archaea, two types of FKBP’s are reported but their relative distribution among archaea from different phyla has not been studied. So, the current study focused on the distribution and occurrence of homologs of different PPIase families in archaea.

Hundred ninety-six Genomes were selected for the current study. The 196 archaeal genomes were further classified into phylum, class and order (Supplementary Table 1).

### Distribution of Cyclophilin Protein Among Archaeal Organisms

**FIGURE 1:**
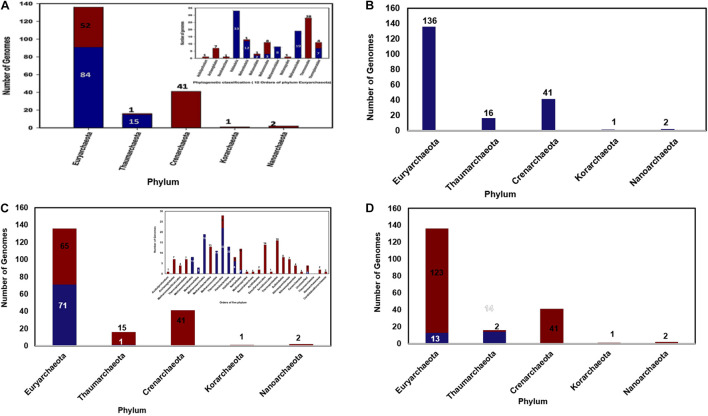
**(A)** Distribution of archaeal cyclophilin protein in 196 archaeal genomes (in 5 phyla), **(B)** Distribution of archaeal long-type FKBP distribution 196 archaeal genomes (in 5 phyla), **(C)** Short-type FKBP distribution, inset shows the distribution of short type FKBP sequences in different class of archaea, **(D)** Distribution of archaeal Parvulin proteins in 196 archaeal genomes (in 5 phyla).

From the constructed database of predicted proteomes of 196 archaeal organisms a total of 122 cyclophilin homologs were identified. From the above sequences it was observed that some cyclophilins were present as multi domain protein and pro-isomerase superfamily domain was found at the N-terminus. After the data curation only 106 sequences of cyclophilin were included in further analysis. On classification of these sequences, it was observed that out of these 106 sequences 91 cyclophilin sequences are derived from 84 organisms of Euryarchaeota and 15 are derived from organism of Thaumarchaeota (Figure 1A).

Several orders of Euryarchaeota i.e., Aciduliprofundum, Archaeoglobales, Nanohaloarchaea, Methanopyrales, and Thermococcales completely lack the cyclophilin gene (Figure 1A). All organisms of order Halobacteriales, Methanomicrobiales and Methanosarcinales possess the cyclophilin gene. Cyclophilin gene is absent from *Candidatus caldiarchaeum subterraneum* organism of phylum Thaumarchaeota.

### Distribution of FK506 Binding Protein Among Archaeal Organisms

Total of 325 archaeal FKBP sequences were extracted from 196 archaeal genomes. It was observed that phylum Euryarchaeota has maximum number of FKBP’s belonging to both long-type and short-type. In 136 genomes of phylum Euryarchaeota, there are about 10 classes and 15 orders, having 264 homologs of FKBP. Hence, it can be assumed that this phylum has maximum diversity in number of FKBP’s homologs present in each organism. It was observed that order Aciduliprofundum, Archaeoglobales, Methanomassiliicoccales, Thermoplasmatales, Methanobacteriales, Methanopyrales, Nanohaloarchaea has single copy of long-type FKBP with no short-type FKBP in their proteomes (Supplementary Table 2). Some of Methanomicrobiales, Thermococcales, Halobacteriales, Haloferacales and most of Natrialbales have single copy of long-type FKBP only. Single copy of each long-type and short-type FKBP is observed in three proteomes of each Methanomicrobiales and Methanosarcinales, nine proteomes of each Methanococcales and Halobacteriales and twenty-two proteomes of Thermococcales. A combination of Single long-type FKBP and multiple short-type FKBPs was observed in sixteen proteomes of Methanosarcinales, four proteomes of each Methanomicrobiales and Haloferacales, two proteomes of each Methanococcales and Halobacteriales, and one proteome of Natrialbales possess (Supplementary Table 2). Multiple copies of each long-type and short type FKBP’s are present in genomes of Methanocellales, and one genome of Natrialbales (Supplementary Table 2). Hence, it was observed that different combination of FKBP’s is highly distributed in phylum Euryarchaeota.

Phylum Crenarchaeota constitutes 41 genomes distributed in class Thermoprotei with its five orders. These 41 genomes have single copy of long type FKBP and complete absence of short-type FKBP (Figures 1B,C). Phylum Thaumarchaeota has 16 genomes distributed in three classes and three orders, and few are unclassified. Nitrosopumilaceae, Nitrososphaerales, and Cenarchaeales consist only single copy of long type FKBP. Among four unclassified genomes, three of them possess only single copy of long-type FKBP while one of unclassified genome has one copy of each long-type and short-type FKBP (Figures 1B,C). Phylum Nanoarchaeota and Korarchaeotahave2 and 1 genome having single copy of long-type FKBP, respectively (Figures 1B,C). From this distribution analysis it was observed that phylum Crenarchaeota, Nanoarchaeota and Korarchaeota have single copy of long-type FKBP and there is no short-type FKBP reported in them. Phylum Thaumarchaeota has 1 short type FKBP along with long-type FKBP.

### Distribution of Parvulin Protein

Total 30 parvulin homologs were found in 196 archaeal organisms (Figure 1D). In phylum Thaumarchaeota single copy of parvulin protein is present in 7, 3, 3, 1 genomes of order Nitrosopumilaceae, Nitrososphaerales, Unclassified Thaumarchaeota and Cenarchaeaceae, respectively. In Euryarchaeota, it is widely distributed in genomes of order Methanomicrobiales, Methanosarcinales, Methanomassiliicoccales, Thermoplasmatales and Nanohaloarchaea (Figure 1D). Multiple copies of parvulin were observed in order Methanomassiliicoccales (*Candidatus Methanomassiliicoccus intestinalis* and *Methanogenic archaeon ISO4-H5*). This distribution shows that parvulin is absent in rest of the phyla.

### Phylogenetic Analyses

#### Phylogenetic Analysis of Archaeal Cyclophilin Proteins

From the phylogenetic tree of the 106 cyclophilin protein sequences, it was evident that the proteins are separated into two main branches. One branch forms the largest cluster which includes cyclophilin homologs from class Halobacteria and second branch has three sub clusters, one having cyclophilins from class Thermoplasmata along with homologs from class Methanomicrobia, second having cyclophilin members from class Methanococci along with homologs from phylum Thaumarchaeota. The third cluster of this branch contains the homologs from class Methanobacteria and Methanomicrobia (Supplementary Figure 2). The bootstrap values obtained from both branches are significantly high which suggests that phylogenetic tree is quite stable.

#### Phylogenetic Analysis of Archaeal FK506 Binding Protein Sequences

In archaea, FKBP’s are reported to be of two types: long-type FKBP and short-type FKBP (Maruyama et al., 2004) in contrast to that found in other organisms. To know the evolutionary differentiation of FKBP in archaea, phylogenetic analysis was constructed with the help of MEGA 6.0. Among the 325 FKBP sequences extracted from the NCBI database; two more sequences were added one Long- type FKBP and one short type FKBP, [of *Methanothermococcus thermolithotrophicus* (whole genome sequence is not annotated)], as it is the only biochemically and genetically characterized organism from archaea (Furutani et al., 1998). So, the total count of FKBP sequences is 327. It was observed that phylogenetic tree of total sequences divides into two clades, long-type and short-type FKBP separated into two branches, constituting 201 and 126 sequences, respectively (Supplementary Figure 3).

Two separate phylogenetic trees were constructed for each long-type and short-type FKBP. In long-type FKBP tree it was observed that it divided into two clades; the FKBP orthologs from organisms of phylum Euryarchaeota and Nanoarchaeota lie in one clade, while those from phylum Crenarchaeota, Thaumarchaeota and Korarchaeota were in the other clade. The clade containing phylum Euryarchaeota, orthologs of class Archaeoglobi and Methanomicrobia lie within the same sub clade of Euryarchaeota, while other orthologs from other classes: Methanopyri, Halobacteria, Thermococci, Methanobacteria, Thermoplasmata, Methanococci and Candidatus Nanohaloarchaeota form a separate sub-clade of each class. In the other branch, orthologs from phylum Thaumarchaeota form a sub clade between phylum Crenarchaeota, while phylum Korarchaeota merged within phylum Crenarchaeota (Supplementary Figure 4).

In short-type FKBP phylogenetic tree, FKBP orthologs from classes of phylum Euryarchaeota was divided in a clade specific manner. There is a single short-type FKBP present in phylum Thaumarchaeota and that lies in the clade with orthologs from Methanomicrobia. This short-type FKBP ortholog from phylum Thaumarchaeota is still unclassified so there lays ambiguity for its classification (Supplementary Figure 5).

#### Phylogenetic Analysis of Archaeal Parvulin Protein Sequences

From the phylogenetic tree of the 30 parvulin protein sequences, it was observed that tree forms two clades, phylum Thaumarchaeota and phylum Euryarchaeota, with an exception of Nanohaloarchaea (*Nanohaloarchaea archaeon SG9*) lying in Thaumarchaeota clade (Supplementary Figure 6). The external nodes have low bootstrap value but internal nodes have a high score which indicates the stability of phylogenetic tree.

### Available Structural Information for Different Peptidyl-Prolyl Isomerase (PDB Structures)

**FIGURE 2:**
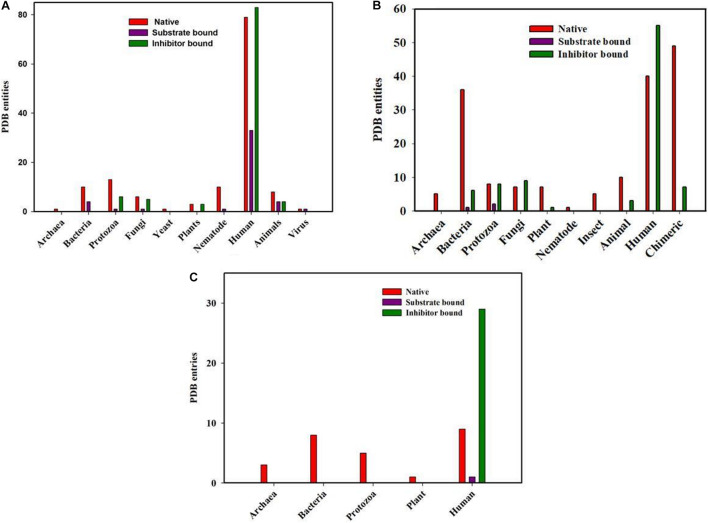
PDB entities of Native, Substrate and inhibitor bound PPIases in RCSB PDB. **(A)** cyclophilin **(B)** FKBP and **(C)** Parvulin from different genera.

To understand the structure function relationship of the proteins of the three classes, the report structures for each member from the RCSB PDB database were collected. It was observed that a total of 300, 294, 71 cyclophilins, FKBPs and parvulin structures are currently available. Most of the available structures come from eukaryotes and bacteria with very few from archaeal organisms (Figure 2).

#### PDB Structures of Cyclophilin Homologs

Total of 300 cyclophilin structures were mined from the RCSB PDB databank with the keyword “cyclophilin.” Out of these 300 PDB entries, 195 structures are for cyclophilin homologs found in Homo sapiens. There are numerous structures which belong to mammals, bacteria, fungi, parasites, yeast, nematode, pathogenic bacteria, plant, virus and archaea. In the available data, 45 and 101 entries are substrate and inhibitor bound (natural as well as synthetic) respectively, whereas the rest are for the native structure. On analyzing the distribution of cyclophilin among bacteria, mammals, plants, viruses, yeast, fungi, nematodes and protozoa it was found that total 14, 16, 6, 2, 1, 12, 11, and 20 structures are reported, respectively, from each group of organisms. Only a single structure is reported from archaea, as the hypothetical protein with cyclophilin like fold from *Archaeoglobus fulgidus* DSM 4304 (Figure 2A).

#### PDB Structures of FK506 Binding Protein Homologs

Total of 294 structures of FKBP proteins from various organisms are deposited in RCSB PDB. Among them 124, 46, 18, 18, 13, 8, 5, 5, 1, and 56 structures belong to human, bacteria, fungi, protozoa, animal, plants, archaea, insects, and nematode, respectively, whereas structures of 56 Chimeric proteins are also included. Out of these available structures 119 are in native form, 3 and 89 are structures of FKBP protein bound to substrate and inhibitor, respectively (Figure 2B).

#### PDB Structures of Parvulin Homologs

In parvulin, total 71 structures have been reported in RCBS PDB. Among them 54, 8, 5, 3, 1 belongs to human, bacteria, protozoa, archaea and plant, respectively. Parvulin is a class of PPIase which is inhibited by juglone. In human out of 54 structures, 9 are native, 29 and 1 are inhibitor and substrate bound, respectively, while 15 structures are reported with mutation. The parvulin structures belonging to bacteria, archaea, protozoa and plants are reported only in their native form with no inhibitor or substrate bound structures have been reported yet (Figure 2C).

### Structural Analysis of Cyclophilin Protein

#### Prediction of Conserved Motifs in Archaeal Cyclophilin Protein

**FIGURE 3:**
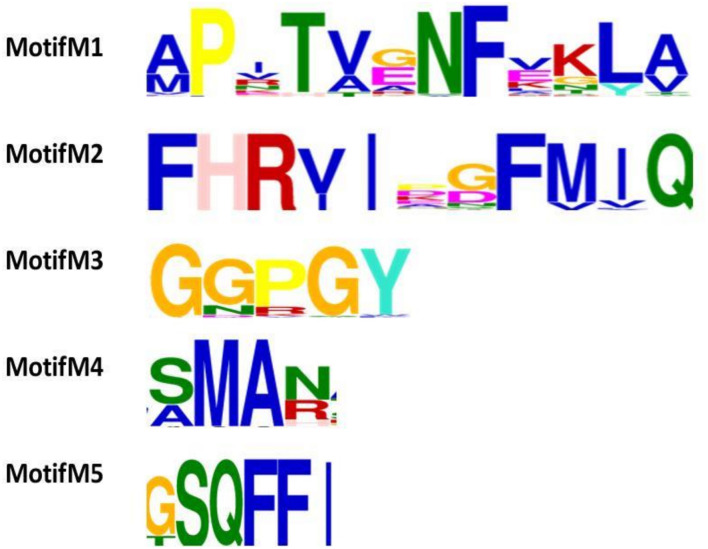
Functional motif analysis in 106 cyclophilin sequences. Five putative motifs M1, M2, M3, M4, and M5 were identified by MEME. The height of the bold letters in motifs represents the relative level of conservation of a particular residue at a given site in the sequences.

The final 106 cyclophilin protein sequences were subjected to Motif predictions. The MEME tool predicts five conserved motifs. Motif M1 consists of an N-terminal motif “AXXTXXNF.” Motif M2 consists of a highly conserved stretch of amino acids with the sequence “FHRV/II.” This motif contains several residues like R_45_, F_50_, V_51_, and Q_53_ that are known to be a part of the active site region. Motif M3 “GXGGXGY” is observed in the mid-region of the sequence. The observed motif appears to be variable in archaeal cyclophilins with only the glycine amino acid is being highly conserved in all the archaeal organisms. Human cyclophilins isoforms had revealed that the substrate binding region of each protein has two pockets namely i.e., S1 and S2 pockets. S1 pocket is shown to be specific for recognizing the proline (P1) residue of the substrate, whereas the S2 pocket creates space for interactions with the substrate residues P2 or P3. Motif M4 contains two highly conserved residues, M_87_ and A_88_, latter being part of the S1 cavity. Motif M5 has the conserved C-terminal motif “GSQFFI” having F_100_, H_108_, L_109_ residues that are known to participate in the formation of S1 cavity of the protein (Figure 3).

#### Comparison of Substrate and Inhibitor Binding Cavity

To examine the effects of inhibitor binding to the cavity of cyclophilin protein, the substrate/inhibitor bound protein structures from different species were compared. In the PDB database, human cyclophilin structures are most abundant. So, the native binding cavity of human Cyp with its inhibitor bounded structures was compared to the other groups. It was found that upon binding of different inhibitors to the human Cyp there is no significant difference in the cavity size and shape. The structures of cyclophilin protein from different species like fungi, protozoa, nematode, plant and bacteria were also compared with native to their inhibitor bound structures. The study reveals that for all the structures from different species, no major differences in the cavity can be seen. However, a single histidine residue shows some fluctuations, between the native and inhibitor bounded state. Human cyclophilin is the most studied one, so we have taken this as a reference to predict the active sites residues among all groups. The active site of the cyclophilin family is known to include the invariant catalytic arginine (Arg55) and a highly conserved mixture of hydrophobic, aromatic, and polar residues including Phe60, Met61, Gln63, Ala101, Phe113, Trp121, Leu122, and His126. The basic residues, R55 and H126, are clearly involved in catalytic acceleration. The structure of hCyPA bound to tetrapeptide (Kallen et al., 1991) shows that the guanidinium side chain of R55 and the imidazole of H126 are in close proximity to the prolyl ring of the bound substrate.

**FIGURE 4:**
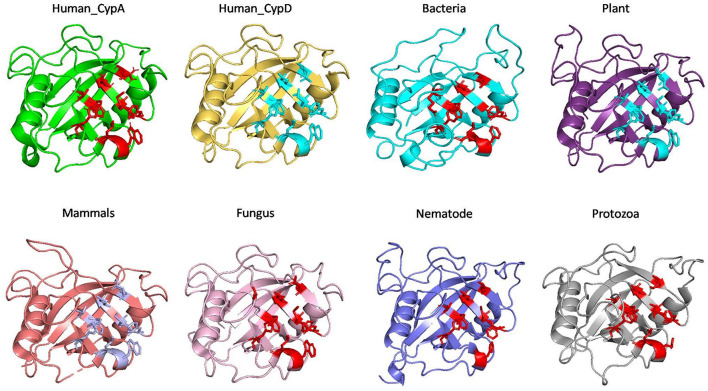
Native structures of cyclophilin from different domains.

**FIGURE 5:**
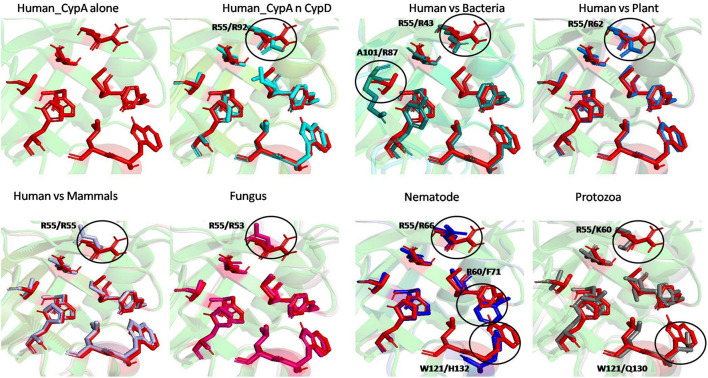
Superimposition of native Human CypA with other native domains. Different orientations of the residues are highlighted by black circle.

On comparing the active site residues of human cyclophilin with orthologs from other organisms, it was observed that orientation of arginine residue at position 55 varies in all the group. In case of cyclophilins from the nematode there is a slight variation at position 60 and 121 (Figures 4, 5).

**FIGURE 6:**
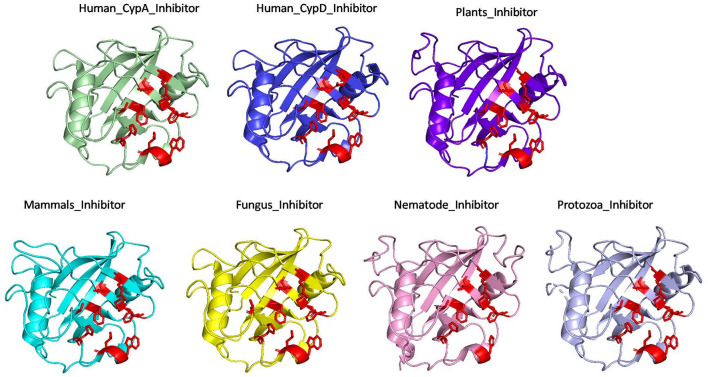
Inhibitor bound structures of cyclophilins from different domains.

**FIGURE 7:**
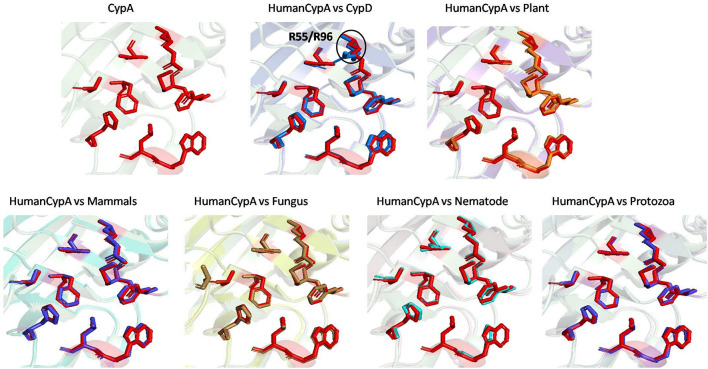
Superimposition of Inhibitor bound human cyclophilin with orthologs from other organisms. Difference in the orientations of the residues is highlighted by black circle. The amino acids depicted in red represent the active sites of Human Cyp in inhibitor bound state.

The effect of inhibitor binding on the cavity in each class was also studied. It was observed that there is no significant difference in the cavity region after binding with inhibitor (Figures 6, 7).

### Structural Analysis of FK506 Binding Protein Proteins

#### Prediction of Conserved Motifs in FK506 Binding Protein Archaeal Protein Sequences

**FIGURE 8:**
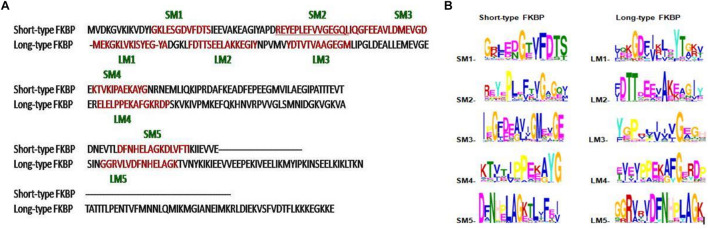
Multiple sequence alignment and Motif analysis of short-type and long-type FKBP from *Methanothermococcus thermolithotrophicus*. Motifs are highlighted in red on sequences along with number **(A)**. SM and LM denotes short-type and long-type FKBP predicted motif **(B)**.

Long-type and short-type FKBP archaeal protein sequences were separately analyzed to know the conservation of residues among the sequences through MEME suite. The hydrophobic active site pocket of archaeal FKBP protein is already known from the previous studies (Suzuki et al., 2003). The dataset of long-type FKBP (201 sequences) was used to analyze the pattern of conserved amino acids, through motifs in archaeal organisms. After analyzing all the five motifs in long type FKBP’s (LM1 to LM5) it was observed that motif LM1 and LM2 contain the active site residues Y_15_, F_25_, D_26_ (Suzuki et al., 2003) which are reported to play an essential part in the substrate binding. Apart from this, motif LM2 also has a small conserved stretch “DTTXXXXA.” Motif LM3 and LM4 are unique and contains glycine as a highly conserved residue whose functional significance and conservation hasn’t been explored yet. In motif LM5, L_138_, L_143_ residues are present, both the Leucine residues are reported to play an important role in substrate binding site. These leucine residues are surrounded by some consensus residues as “DFNHXXXAG” (Figure 8A).

To determine the conserved stretch/residues among the short-type FKBP, 126 short type FKBPs protein sequences were subjected to the MEME. Five predicted motifs possess various residues of hydrophobic-substrate binding site. Motif SM1 consists of “GXXFDTS” where the F_25_ residue is involved in substrate binding site (Suzuki et al., 2003). Motif SM2 consist of many residues from the substrate binding sites i.e., L_48_, F_50_, Q_56,_ L_57_. Motif SM3 consists of I_58_, F_61_ and this motif also possess glycine as conserved residues but the exact function of glycine is not known. Motif SM4 contains active site residue Y_84_ and conserved glycine is present adjacent to tyrosine. Motif SM5 have a small stretch of “DXNXXLAG” and L_138_, L_143_, F_145_ residues which acts as substrate binding sites (Figure 8B).

#### Comparison of Substrate and Inhibitor Binding Cavity

Substrate binding cavity of FKBP proteins is mainly formed by hydrophilic edges and hydrophobic bottom and wall (Ikura and Ito, 2007). Human FKBP12 is the most studied FKBP so far, so it was taken as a reference for determination of substrate binding cavity for all the groups. Substrate binding cavity of FKBP12 includes Y26, F36, D37, R42, F46, V55, I56, W59, Y82, H87, and F99 (Van Duyne et al., 1993). Among these residues D37, R42, F46, V55, W59, and Y82 are known to play important role in PPIase activity (Wilson et al., 1995). Y26, F36, D37, V55, W59, and Y82 are highly conserved amino acid residues in all domains (Furutani et al., 1998).

The archaeal FKBP family includes two types of members: small size FKBP family members contain only FK506-binding domain, while FKBPs with large molecular weight possess extra domains for various other functional activities; mTOR binding site, calcium binding domain (Kang et al., 2008). FKBP52 belongs to large molecular weight category of FKBP and it is composed of three domains: FK506-binding domain, an FKBP-like domain and TRP-clamp domain (Bracher et al., 2013). While, FKBP25 belongs to small size FKBP family and it is consisting of FK506-binding domain at its C-terminal (Prakash et al., 2016).

**FIGURE 9:**
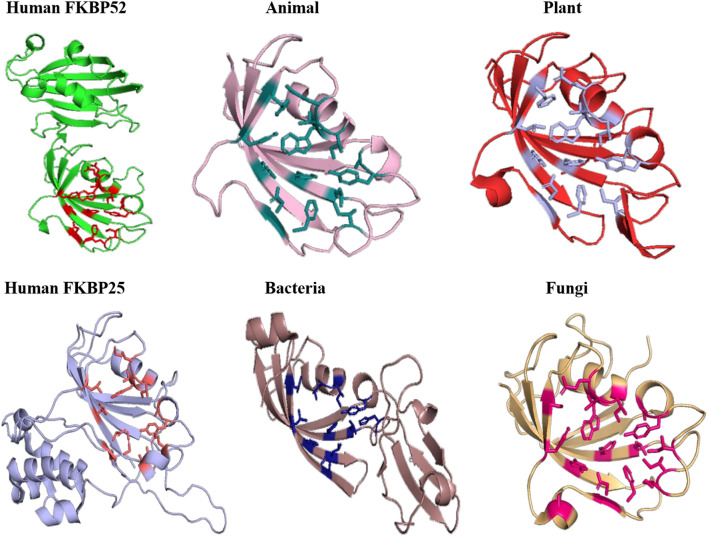
Native structures of FKBP from different domains.

**FIGURE 10:**
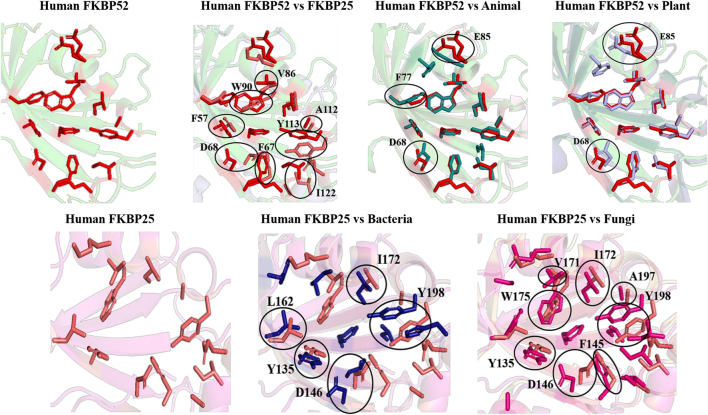
Superimposition of native Human FKBP52 and FKBP25 with other native domains. Different orientations of the residues are highlighted by black circle.

On comparing the orientation of active sites of Human FKBP52 and Human FKBP25 with others it was observed that FK506 binding domain of FKBP52 aligns with human FKBP25, animal and plant only, while bacteria and fungi homologs align with FKBP25 binding domain. FKBP binding domain of protozoa does not align with any of human FKBP’s and this differentiates it from others. On comparison of FK506 binding domain in human (FKBP52 and FKBP25) it was observed that there is variation in orientation of following residues; Y57/Y135, F67/F146, D68/D146, V86/V171, Y113/Y99, while at F77 is replaced by a different residue L162 in FKBP25 (Figures 9, 10).

On comparison of animal and plant with FKBP52, it was observed that there are orientation differences in two substrate binding sites; D37/D48 and E54/E65 (highly conserved residue). F46 in animal as compared to F77 in FKBP52 has different orientation in animal and in plants phenylalanine is replaced by leucine. The above observation shows that substrate binding sites residues are same but there is difference in the orientation of the amino acid residues.

On comparison of human FKBP25 with bacteria and fungi, it was observed that there is a difference in orientation of many catalytic residues; Y135 FKBP25/Y13 bacteria/I60 fungi, D146 FKBP25/D123 bacteria/D41 fungi, I172 FKBP25/I37 bacteria/I60, A195 FKBP25/A62 bacteria/A96fungi and Y196 FKBP25/Y63 bacteria/Y97 fungi. When F145, V171 in FKBP25 is compared with fungi and bacteria it was observed that there is a difference in the orientation of amino acid residue at position F40, V59 in fungi while it is replaced by a different amino acid L15, L36, respectively, in bacteria. Tryptophan in FKBP25 (W175) is an important active site residue but in bacteria it is replaced by L40 while in rest of the domain this residue remains conserved (Figure 10).

**FIGURE 11:**
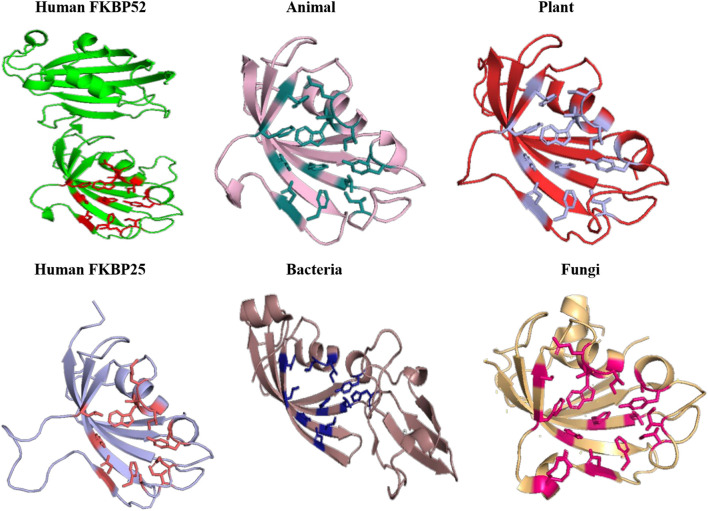
Inhibitor bound structures of FKBP from different domains.

**FIGURE 12:**
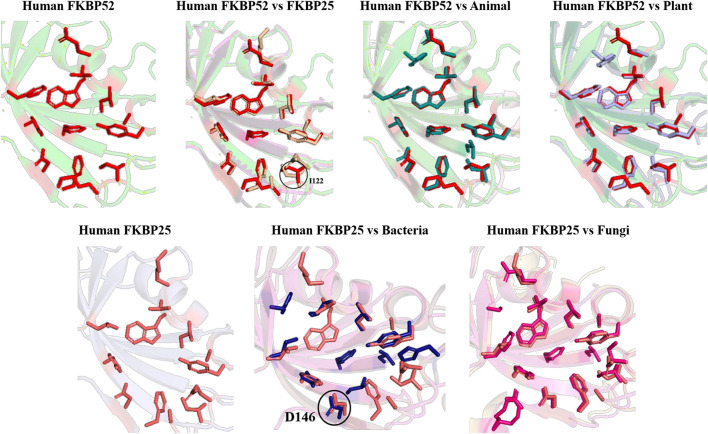
Superimposition of Inhibitor bound human FKBP’s with other domains. Difference in the orientations of the residues is highlighted by black circle. Red and deep salmon represents the active sites of Human FKBP52, FKBP25, respectively with inhibitor bound.

When inhibitor binds to its FKBP52 and 25, it was observed that there are no significant changes in the orientation of active sites residues. Comparison of orientation of active site residues after binding of inhibitor it was observed that all the domains attain an ordered conformation as FKBP52 and FKBP25 (Figures 11, 12).

### Structural Analysis of Parvulin Proteins

#### Prediction of Conserved Motifs in Archaeal Parvulin Protein Sequences

**FIGURE 13:**
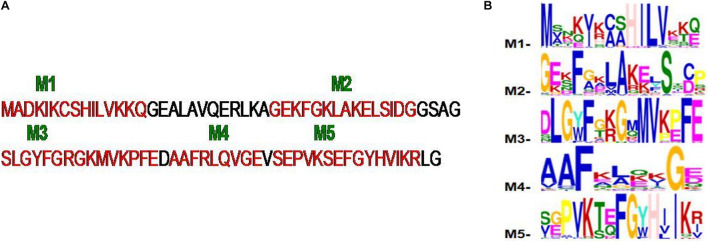
**(A,B)** The five motifs (M1–M5) predicted for archaeal parvulin and represented on *Cenarchaeum symbiosum* sequence.

To explore the conserved amino acid residues in parvulin protein sequences, dataset of 30 sequences was subjected to MEME suite. The already known substrate binding sites of parvulin are H_9_, D_41_, M_59_, F_63_, F_83_, and H_86_ (Jaremko et al., 2011). In five predicted motifs, Motif M1 has “HILV” as consensus stretch and H_9_ is known to be active binding site residue. Motif M2 has D_41_ as active site residue and surrounded by many other conserved residues i.e., phenylalanine, alanine and serine. Motif M3 comprises a small stretch of “MVXXFE” and within this M_59_, F_63_ act as active sites residues. Motif M4 comes out to be unique with conserved residues phenylalanine and glycine. Motif M5 has “FGXHXI” and F_83_ and H_86_ participate in active site residues of parvulin. From here it was observed that all the active sites residues fall in the predicted motifs of parvulin archaeal sequence (Figure 13).

#### Comparison of Substrate and Inhibitor Binding Cavity of Parvulin Protein

In human, two homologous of parvulin are reported; human rotamase/peptidyl-prolyl *cis*-*trans* isomerase (Pin1) and parvulin-like human peptidyl-prolyl *cis*/*trans* isomerase (hPar). In Pin1, substrate binding pocket is hydrophobic in nature and mainly formed by F134, M130, L122 amino acid residues. It is reported that PPIase activity is majorly performed by following residues C113, H59, H157, S154 and basic amino acid residues (K63, R68, R69) are responsible for catalytic selectivity of substrate and inhibitor binding (Ranganathan et al., 1997). While, in human parvulin14(hPar14), L82, M90, F94 forms substrate binding cavity and D74, H42, H123, S72 are responsible for PPIase activity (Sekerina et al., 2000). hPar14 does not have catalytic selectivity as in Pin1.

**FIGURE 14:**
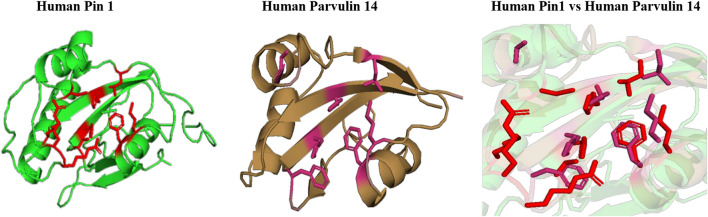
Native structures of parvulin homolog from human and superimposition of active site residues.

On comparing the orientation of active sites of human Pin1 and human Parvulin 14(hPar14) it was observed that same active site amino acid residues (H59/H42, S111/S72, C113/D74, L112/L82, M130/M90, F134/F94, T152/T118, and H157/H123) have different orientation and this could be responsible for the catalytic selectivity of both the protein against substrate and inhibitor (Figure 14).

**FIGURE 15:**
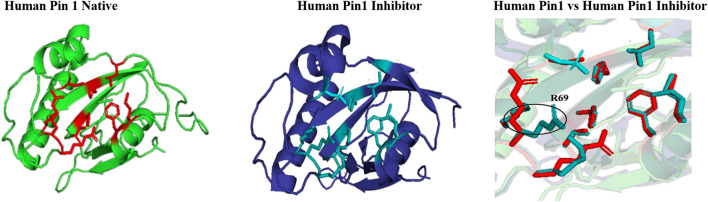
Native and inhibitor bound structure of human Pin1.Superimposition of inhibitor bound human Pin1 with native structure. Difference in the orientations of the residues is highlighted by black circle. Red represents the active sites of Human Pin1 native structure.

The effect of inhibitor binding on the cavity in human Pin1 was also studied. It was observed that there is no significant difference in the cavity region after binding with inhibitor as compared to native Pin1 (Figure 15).

## Discussion

This study is one of it is kind which has the compilation of archaeal PPIases family. This study contributes to distribution of archaeal PPIase in five phyla, phylogenetic evolution, conservation of functional amino acids residues, and comparison of structural variation between native and substrate/inhibitor bound state. It is known that kingdom archaea are classified into five phyla namely: Euryarchaeota, Thaumarchaeota, Crenarchaeota, Nanoarchaeota and Korarchaeota while PPIase family is characterized into three: cyclophilin, FKBP and parvulin. From the 196 collected genomes 136, 41, 16, 2 and 1 genome belong to phylum Euryarchaeota, Crenarchaeota, Thaumarchaeota, Nanoarchaeota and Korarchaeota, respectively (Supplementary Figure 1). These genomes were further classified into classes and order (Supplementary Table 1) for better understanding of distribution. From the proteome database of cyclophilin, its distribution in archaea was studied. On analyzing the available data, it was observed that 196 genomes have 106 cyclophilin protein sequences distributed only in two phyla: Euryarcheota and Thaumarchaeota and most of the organisms have single copy. Most of cyclophilin homologs found in archaea have a domain of PpiB type, and is classified as separate domain family as compared to the cyclophilin domain found in eukaryotes and bacteria, suggesting that the cyclophilin domains in the three kingdoms have evolved in separate directions. On studying the distribution of FKBP from FKBP proteome database, it was analyzed that 196 genomes have 325 archaeal FKBP sequences. A higher number of proteins compared to the genomes suggests that organisms may have multiples copies of FKBP’s. It was earlier reported that archaeal FKBP are of two types: long and short type. To know how they are distributed, the organisms were classified on the basis of number of paralogs of long-type and short-type FKBP’s. Each archaeal organism has one of the following combinations; only single copy of long-type, single copy of each long-type and short-type, single copy of long-type and multiple copies of short-type, and multiples copies of both long-type and short-type (Supplementary Material). Further analysis also reveals that long-type FKBP are present in all five phyla in 196 genomes, while short type is present in Euryarcheota (except DHVE2 Group, Archaeoglobi, Thermoplasmata, Methanobacteria, Methanopyri, Candidatus Nanohaloarchaeota classes) and Thaumarchaeota. Crenarchaeota, Nanoarchaeota and Korarchaeota phyla have only long-type FKBP’s (Supplementary Table 2). In conserved domain analysis it was found that FKBP in archaea belongs to SlpA superfamily while FKBP’s in bacteria are reported to be of different types namely FkpA, FkpB, SlyD, trigger factors, Mip-type (Baneyx and Mujacic, 2004). Third family of PPIase, parvulin is infrequently present, except in 13 and 14 genomes of Euryarcheota and Thaumarchaeota, respectively (Supplementary Table 3). This number is much lesser than the number of genomes, hence there are lesser organisms in archaeal kingdom which have parvulin. On analyzing its conserved domain, it was inferred that they belong to rotamase2 superfamily which mostly have PPIC type PPIase domain, which differentiate them from bacterial parvulin which are SurA, PpiD type (Ünal and Steinert, 2014). Above analysis reveals that cyclophilins, short-type FKBP and parvulin are present in phylum Euryarchaeota and Thaumarchaeota only, while Crenarchaeota, Nanoarchaeota and Korarchaeota have long-type FKBP only. Hence, it could be concluded that in these three phlya (Crenarchaeota, Nanoarchaeota and Korarchaeota) PPIase function is performed by long-type FKBP along with chaperone function while it might be shared between different types of PPIases in other two phyla. Till now PPIase in archaea are known to have both PPIase and chaperone like activity while periplasmic PPIase in *E. coli* are reported to exert their function mainly through chaperone like activity (Stull et al., 2018). Hence, it can be postulated that PPIases can perform both the functions (PPIase and chaperone activity) in some organisms while it can perform only one type of function in other organisms. In Phylogenetic analysis of PPIases, it was observed in cyclophilin that there is mixing of Euryarcheota and Thaumarchaeota phylum, while in FKBP and parvulin they form separate clade of each phylum. In FKBP, long-type and short-type forms two separate clades. Halobacteria order of Euryarchaeota forms a separate clade in the phylogenetic tree of both cyclophilin and FKBP, while parvulin is absent in halophiles. And, there is mixing of Methanomicrobia with other orders in both cyclophilin and long-type FKBP. In short-type FKBP, there is no mixing of orders, hence they separate in clade specific manner (Supplementary Figures 3–6). From the phylogenetic analysis it could be suggested that long-type and short-type FKBP have evolved separately.

To explore the conservation of functional residues and structural variation in each class of PPIases, motifs were predicted and structural analysis were performed. Most of the reported PDB structure are from human in all the three classes. Among reported PDBs, substrate bound structures are few in number, while inhibitor bound structures have been reported more among all three classes of PPIases (Figures 2A–C). PDB structures reported in bacteria in all three classes are very less in number as compared to those reported from humans. Moreover, PDB structures reported in all three classes are either substrate bound (cyclophilin), or inhibitor and substrate bound (FKBP), or only native (parvulin). Hence, this made it difficult to compare the structural transitions among the three classes of PPIases. Predicted motifs in cyclophilin suggests that most of functional amino acid residues lie within these predicted motifs along with highly conserved glycine (Motif M3) (Figure 3). As human cyclophilin is most widely characterized, it was taken as reference to study structural variation. On superimposition of native human cyclophilin with homologs from other organisms it was observed that there are some variations in amino acids residues of active site (Figure 5). However, when the inhibitor bound structures were superimposed, all homologs display the same conformation as human cyclophilin (Figure 7).

In long-type and short-type FKBP homologs, the predicted motifs overlap with each other when compared (Figure 8A) and suggest that most of the functionally important residues lies in the N-terminal of long-type FKBP, while C-terminal lacks any conservation of amino acid residues. It has been reported that C-terminal of fkpA in *E. coli* have PPIase domain and N-terminal has dimerization and chaperone activity, while in archaeal FKBP PPIase domain is present at N-terminal and the function of the extra C-terminal region remains undiscovered (Saul et al., 2004). The predicted conserved glycine residues are not yet explored, which might be contributing toward some structural stability or functional flexibility to these proteins. For comparison of structural variation human FKBP12 (which is most studied FKBP) was taken as reference for comparing active site residues. It is reported that FKBPs are of two types: one having only FKBP domain and other having FKBP domain along with other domains. So, we have taken each representative from each human FKBP25 and human FKBP52, respectively. On detailed analyses it was observed that there is high variation in active site residues and conformation among all groups in their native state (Figure 10). On comparing inhibitor bound structures it was observed that they align to attain the same conformation (Figure 12).

In parvulin, predicted motifs have most of the functionally important residues (Figure 13). As there are two reported structure one of human Pin1 and human parvulin 14, both were taken to study the variation within parvulin. It was observed that catalytic residues remain same but their orientation varies in both in their native state (Figure 14). Inhibitor bound structure was available for human Pin 1 only, so human Pin 1 native and inhibitor bound structures were compared. On superimposition, it was observed that all catalytic residues attain same conformation as in native state except one amino acid residue i.e., R69 (Figure 15). These inferences suggests that binding pocket forming residues remain almost same in all groups in all classes of PPIases. And binding of respective inhibitor in cyclophilin and FKBP might be driving all studied groups to attain same conformation. Recent studies regarding Pin1 indicate that it can also acts as a molecular timer to help control the amplitude and duration of cellular process in phosphorylation dependent and independent manner (Lu et al., 2007). However, their limited presence in archaeal organisms raises the question of their actual role in the viability of these organisms.

This study highlights the distribution of archaeal PPIase and how the three classes in various phylum may share their function. It was also predicted that long type FKBP may be the main contributors of the PPIase function and chaperone activity as they are ubiquitous in archaea. Over the course of evolution, several differences between the archaeal FKBP in comparison to those form Bacteria and eukaryotes have appeared. For example, in archaea, the PPIase domain is present at N-terminal in FKBP while it is at C-terminal in bacteria. Conserved domain analysis also reveals that PPIase domain in archaea are different enough from those found in homologs of eukaryotes and bacteria, to be classified as different domain family. The function of several residues, like the conserved glycine residue remains ambiguous as its function or contribution remain undiscovered despite being conserved in both archaeal cyclophilin and FKBP. Beside some change in active site residues in all groups in all the three classes, each of them attains same inhibitor bound structure, inferring that PPIase are in more stable conformation when bound to their inhibitors.

## Conclusion

Peptidyl-Prolyl Isomerase play a vital role in various cellular functions, helping in refolding of protein under harsh or stress condition and stabilization of protein during intracellular transport. PPIases function as an accelerating agent of the *cis*-*trans* interconversion (Nath and Isakov, 2015). PPIase family is highly conserved in three domains i.e., Eukaryotes, Bacteria, and archaea (Maruyama and Furutani, 2000; Galat, 2003). Eukaryotes and bacteria have multiple copies of various PPIase e.g., *Saccharomyces cerevisiae* have 8 cyclophilins, 4 FKBPs, and 2 parvulins copies whereas in humans have 18 cyclophilins and 16 FKBPs copies are present (Arevalo-Rodriguez et al., 2004; Erlejman et al., 2013). PPIase in eukaryotes and bacteria have been explored for their immunomodulatory and virulence property, respectively, while in archaea their physiological role remains undiscovered. This study is an attempt to fill a gap of information between archaea and other two domains (eukaryotes and prokaryotes). Classification of archaeal proteome will lay the foundation to known the presence of various PPIases in single organism and which of them is majorly present and how they share their function in others. Phylogenetic analysis reveals that archaeal PPIases are distributed in class specific manner. Most of the catalytic residues are part of predicted motifs with some other conserved residues whose role has not being studied yet. On analyzing the catalytic sites of PPIases it was observed that orientation of active site residues may vary in its native state in different domains but while binding to inhibitor they all adopt the same orientation. On comparison of reported structures in its native and inhibitor bound form it was observed that in homologs from organisms of all kingdom, inhibitor bound structures attain same conformation. In eukaryotes, along with FKBP domain other domains are also present which help in performing various other functions like signal transduction, calcium binding etc. Hence, it can be concluded that the function of PPIase may vary from eukaryotes, bacteria and archaea. The PPIases in eukaryotes and bacteria have evolved and found use in cellular physiology beyond their PPIase and chaperone like activity, but similar role for archaeal homologs remains to be established.

## Data Availability Statement

The datasets presented in this study can be found in online repositories. The names of the repository/repositories and accession number(s) can be found in the article/Supplementary Material.

## Author Contributions

VK, Anchal, and MG planned the whole study, analyzed the data, and wrote the manuscript. VK and Anchal compiled the work. All authors contributed to the article and approved the submitted version.

## Conflict of Interest

The authors declare that the research was conducted in the absence of any commercial or financial relationships that could be construed as a potential conflict of interest.

## Publisher’s Note

All claims expressed in this article are solely those of the authors and do not necessarily represent those of their affiliated organizations, or those of the publisher, the editors and the reviewers. Any product that may be evaluated in this article, or claim that may be made by its manufacturer, is not guaranteed or endorsed by the publisher.

## Abbreviations

PPIase: Peptidyl-prolyl isomerase
CyP: Cyclophilin
CsA: cyclosporine A
FKBP: FK506 binding protein.

## References

[B1] Arevalo-Rodriguez M. Wu X. Hanes S. D. Heitman J. (2004). Prolyl isomerases in yeast. *Front. Biosci.* 9:2420–2446. 10.2741/1405 15353296

[B2] Bailey T. L. Boden M. Buske F. A. Frith M. Grant C. E. Clementi L. (2009). MEME SUITE: tools for motif discovery and searching. *Nucleic Acids Res.* 2009 W202–W208. 10.1093/nar/gkp335 19458158 PMC2703892

[B3] Baneyx F. Mujacic M. (2004). Recombinant protein folding and misfolding in *Escherichia coli*. *Nat. Biotechnol.* 22 1399–1408. 10.1038/nbt1029 15529165

[B4] Bracher A. Kozany C. Hähle A. Wild P. Zacharias M. Hausch F. (2013). Crystal structures of the free and ligand-bound FK1-FK2 domain segment of FKBP52 reveal a flexible inter-domain hinge. *J. Mol. Biol.* 425 4134–4144. 10.1016/j.jmb.2013.07.041 23933011

[B5] Brandts J. F. Halvorson H. R. Brennan M. (1975). Consideration of the Possibility that the slow step in protein denaturation reactions is due to cis-trans isomerism of proline residues. *Biochemistry.* 14 4953–4963. 10.1021/bi00693a026 241393

[B6] Erlejman A. G. Lagadari M. Galigniana M. D. (2013). Hsp90-binding immunophilins as a potential new platform for drug treatment. *Future Med. Chem.* 5 591–607. 10.4155/fmc.13.7 23573975

[B7] Fischer G. Wittmann-Liebold B. Lang K. Kiefhaber T. Schmid F. X. (1989). Cyclophilin and peptidyl-prolyl cis-trans isomerase are probably identical proteins. *Nature* 337 476–478. 10.1038/337476a0 2492638

[B8] Furutani M. Iida T. Yamano S. Kamino K. Maruyama T. (1998). Biochemical and genetic characterization of an FK506-sensitive peptidyl prolyl cis-trans isomerase from a thermophilic archaeon, Methanococcusthermolithotrophicus. *J. Bacteriol.* 180 388–394. 10.1128/JB.180.2.388-394.1998 9440528 PMC106894

[B9] Galat A. (2003). Peptidylprolyl cis/trans isomerases (immunophilins): biological diversity–targets–functions. *Curr. Top Med. Chem.* 3 1315–1347. 10.2174/1568026033451862 12871165

[B10] Harding M. W. Galat A. Uehling D. E. Schreiber S. L. (1989). A receptor for the immunosuppressant FK506 is a cis-trans peptidyl-prolyl isomerase. *Nature* 341 758–760. 10.1038/341758a0 2477715

[B11] Hartl F. (1996). Molecular chaperones in cellular protein folding. *Nature* 381 571–580. 10.1038/381571a0 8637592

[B12] Hartl F. U. Hayer-Hartl M. (2002). Molecular chaperones in the cytosol: from nascent chain to folded protein. *Science* 295 1852–1858. 10.1126/science.1068408 11884745

[B13] Hoppstock L. Trusch F. Lederer C. van West P. Koenneke M. Bayer P. (2016). NmPin from the marine thaumarchaeote Nitrosopumilus maritimus is an active membrane associated prolyl isomerase. *BMC Biol.* 14:53. 10.1186/s12915-016-0274-1 27349962 PMC4922055

[B14] Ikura T. Ito N. (2007). Requirements for peptidyl-prolyl isomerization activity: a comprehensive mutational analysis of the substrate-binding cavity of FK506-binding protein 12. *Protein Sci.* 16 2618–2625. 10.1110/ps.073203707 18029417 PMC2222811

[B15] Jaremko L. Jaremko M. Elfaki I. Mueller J. W. Ejchart A. Bayer P. (2011). Structure and dynamics of the first archaeal parvulin reveal a new functionally important loop in parvulin-type prolyl isomerases. *J. Biol. Chem.* 286 6554–6565. 10.1074/jbc.M110.160713 21138844 PMC3057832

[B16] Kallen J. Spitzfaden C. Zurini M. G. Wider G. Widmer H. Wüthrich K. (1991). Structure of human cyclophilin and its binding site for cyclosporin A determined by X-ray crystallography and NMR spectroscopy. *Nature* 353 276–279. 10.1038/353276a0 1896075

[B17] Kang C. B. Hong Y. Dhe-Paganon S. Yoon H. S. (2008). FKBP family proteins: immunophilins with versatile biological functions. *Neurosignals* 16 318–325. 10.1159/000123041 18635947

[B18] Kaushik V. Prasad S. Goel M. (2019). Biophysical and biochemical characterization of a thermostable archaeal cyclophilin from Methanobrevibacterruminantium. *Int. J. Biol. Macromol.* 139 139–152. 10.1016/j.ijbiomac.2019.07.149 31369788

[B19] Kino T. Hatanaka H. Miyata S. Inamura N. Nishiyama M. Yajima T. (1987). FK-506, a novel immunosuppressant isolated from a Streptomyces. II. Immunosuppressive effect of FK-506 in vitro. *J. Antibiot.* 40 1256–1265. 10.7164/antibiotics.40.1256 2445722

[B20] Koonin E. V. (2015). Origin of eukaryotes from within archaea, archaeal eukaryome and bursts of gene gain: eukaryogenesis just made easier? philosophical transactions of the royal society of london. *Ser. B Biol. Sci.* 370:20140333. 10.1098/rstb.2014.0333 26323764 PMC4571572

[B21] Lin C. H. Li H. Y. Lee Y. C. Calkins M. J. Lee K. H. Yang C. N. (2015). Landscape of Pin1 in the cell cycle. *Exp. Biol. Med.* 240 403–408. 10.1177/1535370215570829 25662955 PMC4935233

[B22] Lu K. Finn G. Lee T. Nicholson L. K. (2007). Prolyl *cis-trans* isomerization as a molecular timer. *Nat. Chem. Biol.* 3 619–629. 17876319 10.1038/nchembio.2007.35

[B23] Madeira F. Park Y. M. Lee J. Buso N. Gur T. Madhusoodanan N. (2019). The EMBL-EBI search and sequence analysis tools APIs in 2019. *Nucleic Acids Res.* 47 W636–W641. 10.1093/nar/gkz268 30976793 PMC6602479

[B24] Martinez-Hackert E. Hendrickson W. A. (2011). Structural analysis of protein folding by the long-chain archaeal chaperone FKBP26. *J. Mol. Biol.* 407 450–464. 10.1016/j.jmb.2011.01.027 21262232 PMC3099347

[B25] Maruyama T. Furutani M. (2000). Archaeal peptidyl prolyl cis-trans isomerases (PPIases). *Front. Biosci.* 5:D821–D836. 10.2741/maruyama 10966874

[B26] Maruyama T. Suzuki R. Furutani M. (2004). Archaeal peptidyl prolyl cis-trans isomerases (PPIases) update 2004. *Front. Biosci.* 9:1680–1720. 10.2741/1361 14977579

[B27] Nath P. R. Isakov N. (2015). Insights into peptidyl-prolyl cis-trans isomerase structure and function in immunocytes. *Immunol. Lett.* 163 120–131. 10.1016/j.imlet.2014.11.002 25445495

[B28] Netzer W. J. Hartl F. U. (1998). Protein folding in the cytosol: chaperonin-dependent and -independent mechanisms. *Trends Biochem. Sci.* 23 68–73. 10.1016/s0968-0004(97)01171-7 9538692

[B29] Notredame C. Higgins D. G. Heringa J. T. - (2000). Coffee: A novel method for fast and accurate multiple sequence alignment. *J. Mol. Biol.* 302 205–217. 10.1006/jmbi.2000.4042 10964570

[B30] Ou W. B. Luo W. Park Y. D. Zhou H. M. (2001). Chaperone-like activity of peptidyl-prolyl cis-trans isomerase during creatine kinase refolding. *Protein Sci.* 10 2346–2353. 10.1110/ps.23301 11604540 PMC2374073

[B31] Pahl A. Brune K. Bang H. (1997). Fit for life? Evolution of chaperones and folding catalysts parallels the development of complex organisms. *Cell Stress Chaperones* 1997:2. 10.1379/1466-12681997002 PMC312984 9250398

[B32] Penn O. Privman E. Ashkenazy H. Landan G. Graur D. Pupko T. (2010). GUIDANCE: a web server for assessing alignment confidence scores. *Nucleic Acids Res.* 38 W23–W28. 10.1093/nar/gkq443 20497997 PMC2896199

[B33] Prakash A. Rajan S. Yoon H. S. (2016). Crystal structure of the FK506 binding domain of human FKBP25 in complex with FK506. *Protein Sci.* 25 905–910. 10.1002/pro.2875 26749369 PMC4941220

[B34] Ranganathan R. Lu K. P. Hunter T. Noel J. P. (1997). Structural and functional analysis of the mitotic rotamase Pin1 suggests substrate recognition is phosphorylation dependent. *Cell.* 89 875–886. 10.1016/s0092-8674(00)80273-1 9200606

[B35] Reed C. J. Lewis H. Trejo E. Winston V. Evilia C. (2013). Protein adaptations in archaeal extremophiles. *Archaea* 2013:373275. 10.1155/2013/373275 24151449 PMC3787623

[B36] Saul F. A. Arié J. P. Vulliez-le Normand B. Kahn R. Betton J. M. Bentley G. A. (2004). Structural and functional studies of FkpA from *Escherichia coli*, a cis/trans peptidyl-prolyl isomerase with chaperone activity. *J. Mol. Biol.* 335 595–608. 10.1016/j.jmb.2003.10.056 14672666

[B37] Schmid F. X. (1993). Prolyl Isomerase: enzymatic catalysis of slow protein-folding reactions. *Ann. Rev. Biophys. Biomol. Struct.* 1 123–143. 10.1146/annurev.bb.22.060193.001011 7688608

[B38] Sekerina E. Rahfeld J. U. Müller J. Fanghänel J. Rascher C. Fischer G. (2000). NMR solution structure of hPar14 reveals similarity to the peptidyl prolyl cis/trans isomerase domain of the mitotic regulator hPin1 but indicates a different functionality of the protein. *J. Mol. Biol.* 301 1003–1017. 10.1006/jmbi.2000.4013 10966801

[B39] Stull F. Betton J. M. Bardwell J. C. A. (2018). Periplasmic Chaperones and Prolyl Isomerases. *EcoSal. Plus* 8:18. 10.1128/ecosalplus.ESP-0005-2018 29988001 PMC11575675

[B40] Suzuki R. Nagata K. Yumoto F. Kawakami M. Nemoto N. Furutani M. (2003). Three-dimensional solution structure of an archaeal FKBP with a dual function of peptidyl prolyl cis-trans isomerase and chaperone-like activities. *J. Mol. Biol.* 328 1149–1160. 10.1016/s0022-2836(03)00379-6 12729748

[B41] Tamura K. Stecher G. Peterson D. Filipski A. Kumar S. (2013). MEGA6: molecular evolutionary genetics analysis version 6.0. *Mol. Biol. Evol.* 30 2725–2729. 10.1093/molbev/mst197 24132122 PMC3840312

[B42] Ünal C. M. Steinert M. (2014). Microbial peptidyl-prolyl cis/trans isomerases (PPIases): virulence factors and potential alternative drug targets. *Microbiol. Mol. Biol. Rev.* 78 544–571. 10.1128/MMBR.00015-14 25184565 PMC4187684

[B43] Van Duyne G. D. Standaert R. F. Karplus P. A. Schreiber S. L. Clardy J. (1993). Atomic structures of the human immunophilin FKBP-12 complexes with FK506 and rapamycin. *J. Mol. Biol.* 229 105–124. 10.1006/jmbi.1993.1012 7678431

[B44] Velazquez H. A. Hamelberg D. (2015). Dynamical role of phosphorylation on serine/threonine-proline Pin1 substrates from constant force molecular dynamics simulations. *J. Chem. Phys.* 142:075102. 10.1063/1.4907884 25702031

[B45] Wilson K. P. Yamashita M. M. Sintchak M. D. Rotstein S. H. Murcko M. A. Boger J. (1995). Comparative X-ray structures of the major binding protein for the immunosuppressant FK506 (tacrolimus) in unliganded form and in complex with FK506 and rapamycin. *Acta Crystallogr. D BiolCrystallogr.* 51 511–521. 10.1107/S0907444994014514 15299838

[B46] Wong A. Gehring C. Irving H. R. (2015). Conserved functional motifs and homology modeling to predict hidden moonlighting functional sites. *Front. Bioeng. Biotechnol.* 3:82. 10.3389/fbioe.2015.00082 26106597 PMC4460814

[B47] Zhang X. C. Wang W. D. Wang J. S. Pan J. C. (2013). PPIase independent chaperone-like function of recombinant human Cyclophilin A during arginine kinase refolding. *FEBS Lett.* 587 666–672. 10.1016/j.febslet.2013.01.028 23376614

