# Supplementary material for: Distribution of Peptidyl-Prolyl Isomerase (PPIase) in the Archaea
Source: Front Microbiol. 2021 Oct 7;12:751049. doi: 10.3389/fmicb.2021.751049 (PMC8530231; doi:10.3389/fmicb.2021.751049)
Supplement: Supplementary file 1 [file Data_Sheet_1.docx]

**Distribution of Peptidyl-prolyl isomerase** (**PPIase) in third domain of life: an archaea**

Anchal^1^, Vineeta Kaushik^1,2^, Manisha Goel^1^*

^1^Department of Biophysics, University of Delhi South Campus, New Delhi-110021, India

^2^International Centre for Translational Eye Research, Institute of Physical Chemistry, Polish Academy of Sciences, Warsaw, Poland.

^*^Corresponding authors: Dr. Manisha Goel, Department of Biophysics, University of Delhi South Campus, New Delhi-110021, India, Email id: manishagoel@south.du.ac.in, Contact number: +91 8588839751 *Email address*: [manishagoel@south.du.ac.in](mailto:manishagoel@south.du.ac.in)

**Collection and distribution of PPIase among archaea**

196 gemones were selected for the current study. It was observed that out of 196 archaealgenomes 136, 41,16,2 and 1 genome belong to phylum Euryarchaeota, Crenarchaeota, Thaumarchaeota, Nanoarchaeotaand Korarchaeota respectively (**Figure S1**).


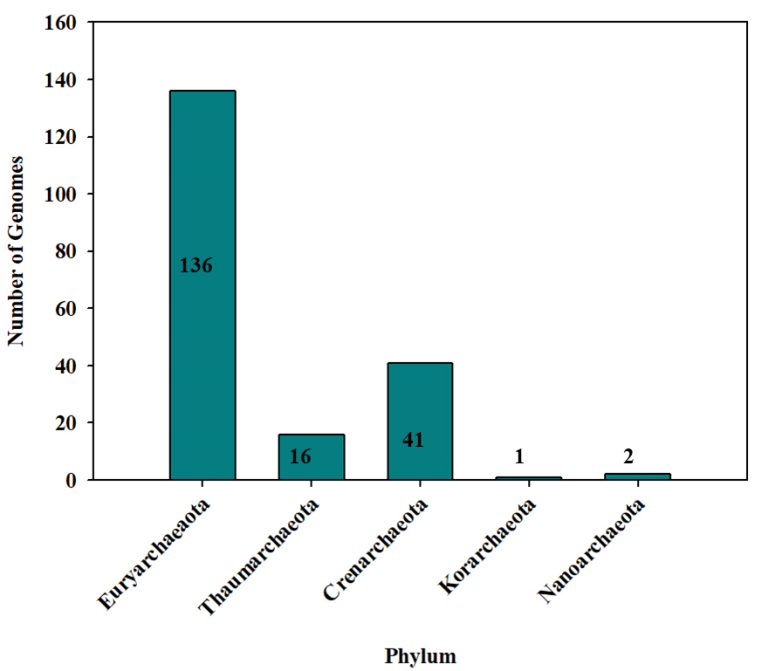


**Figure S1:** Distribution of 196 archaeal genomes.

The obtained genomes were further classified according to their class and orders.

**Table S1: Distribution of archaeal genomes in each class.**

| **S.No** | **Class** | **Order** | **Number of Genomes** |
| --- | --- | --- | --- |
| 1 | DHVE2 Group | Aciduliprofundum | 1 |
| 2 | Archaeoglobi | Archaeoglobales | 7 |
| 3 | Thermoplasmata | Methanomassillicoccales | 4 |
|  |  | Thermoplasmatales | 7 |
| 4 | Methanomicrobia | Methanomicrobiales | 8 |
|  |  | Methanocellales | 3 |
|  |  | Methanosarcinales | 19 |
| 5 | Methanobacteria | Methanobacteriales | 13 |
| 6 | Methanococci | Methanococcales | 11 |
| 7 | Thermococii | Thermococcales | 28 |
| 8 | Halobacteria | Halobacteriales | 13 |
|  |  | Haloferacales | 8 |
|  |  | Natrialbales | 12 |
| 9 | Methanopyri | Methanopyrales | 1 |
| 10 | CandidatusNanohaloarchaeota | Nanohaloarchaea | 1 |
| 11 | Thermoprotei | Acidilobales | 2 |
|  |  | Desulfurococcales | 14 |
|  |  | fervidicoccales | 1 |
|  |  | Thermoproteales | 16 |
|  |  | Sulfolobales | 8 |
| 12 | Nitrosopumilales | Nitrosopumilaceae | 7 |
| 13 | Nitrososphaeria | Nitrososphaerales | 4 |
| 14 | Incertaesedis | Cenarchaeales | 1 |
| 15 | Unclassified  Thaumarchaeota | Unclassified  Thaumarchaeota | 4 |
| 16 | Nanoarchaeales | Nanoarchaeaceae | 2 |
| 17 | CandidatusKorarchaeota | CandidatusKorarchaeota | 1 |

**Table S2: Distribution of archaeal FKBP protein in each class.**

| **S.No** | **Class** | **Order** | **Long type FKBP’s** | **Short type FKBP’s** | **Total** |
| --- | --- | --- | --- | --- | --- |
| 1 | DHVE2 Group | Aciduliprofundum | 1 | 0 | 1 |
| 2 | Archaeoglobi | Archaeoglobales | 7 | 0 | 7 |
| 3 | Thermoplasmata | Methanomassillicoccales | 4 | 0 | 4 |
|  |  | Thermoplasmatales | 7 | 0 | 7 |
| 4 | Methanomicrobia | Methanomicrobiales | 8 | 14 | 22 |
|  |  | Methanocellales | 6 | 5 | 11 |
|  |  | Methanosarcinales | 19 | 44 | 63 |
| 5 | Methanobacteria | Methanobacteriales | 13 | 0 | 13 |
| 6 | Methanococci | Methanococcales | 11 | 13 | 24 |
| 7 | Thermococii | Thermococcales | 28 | 22 | 50 |
| 8 | Halobacteria | Halobacteriales | 13 | 13 | 26 |
|  |  | Haloferacales | 8 | 10 | 18 |
|  |  | Natrialbales | 13 | 3 | 16 |
| 9 | Methanopyri | Methanopyrales | 1 | 0 | 1 |
| 10 | CandidatusNanohaloarchaeota | Nanohaloarchaea | 1 | 0 | 1 |
| 11 | Thermoprotei | Acidilobales | 2 | 0 | 2 |
|  |  | Desulfurococcales | 14 | 0 | 14 |
|  |  | fervidicoccales | 1 | 0 | 1 |
|  |  | Thermoproteales | 16 | 0 | 16 |
|  |  | Sulfolobales | 8 | 0 | 8 |
| 12 | Nitrosopumilales | Nitrosopumilaceae | 7 | 0 | 7 |
| 13 | Nitrososphaeria | Nitrososphaerales | 4 | 0 | 4 |
| 14 | Incertaesedis | Cenarchaeales | 1 | 0 | 1 |
| 15 | Unclassified  Thaumarchaeota | Unclassified  Thaumarchaeota | 4 | 1 | 5 |
| 16 | Nanoarchaeales | Nanoarchaeaceae | 2 | 0 | 2 |
| 17 | CandidatusKorarchaeota | CandidatusKorarchaeota | 1 | 0 | 1 |
| Total | | | | | 325 |

**Table S3:** Distribution of archaeal Parvulin proteins in classes

| **S.No** | **Class** | **Order** | **Number of Genomes** |
| --- | --- | --- | --- |
| 1 | Thermoplasmata | Methanomassillicoccales | 4 |
|  |  | Thermoplasmatales | 1 |
| 2 | Methanomicrobia | Methanomicrobiales | 5 |
|  |  | Methanosarcinales | 2 |
| 3 | CandidatusNanohaloarchaeota | Nanohaloarchaea | 1 |
| 4 | Nitrosopumilales | Nitrosopumilaceae | 7 |
| 5 | Nitrososphaeria | Nitrososphaerales | 3 |
| 6 | Incertaesedis | Cenarchaeales | 1 |
| 7 | Unclassified  Thaumarchaeota | Unclassified  Thaumarchaeota | 3 |


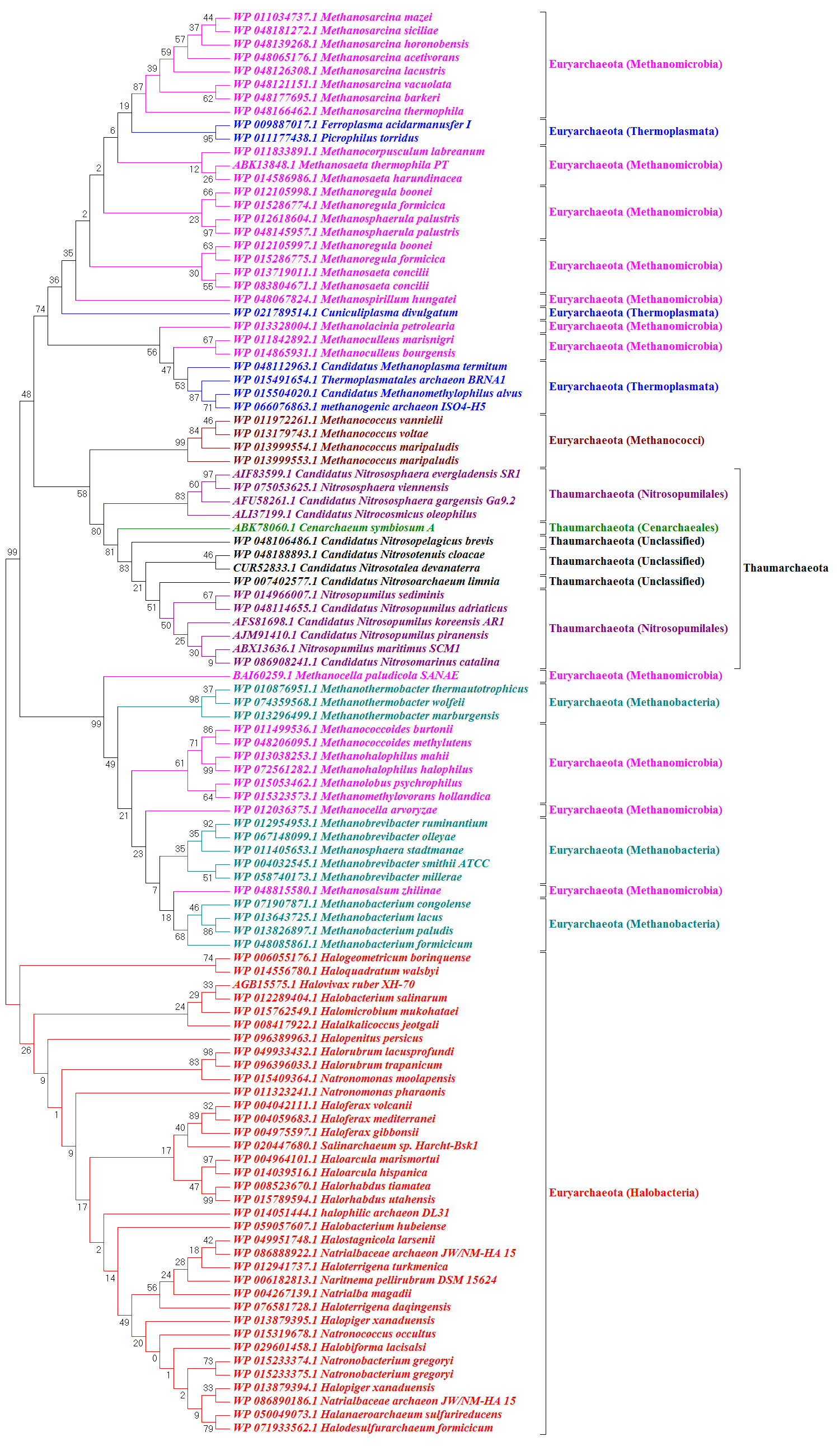


**Figure S2**: Phylogenetic tree of 106 cyclophilin sequences.


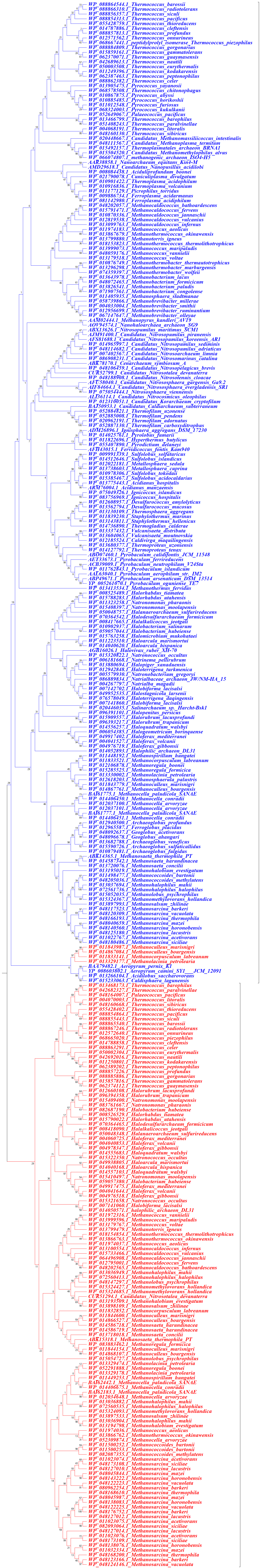


**Figure S3**: Phylogenetic tree of archaeal FKBP. The tree of 327 sequences divides into two clades: long-type and short-type FKBP highlighted in blue and red.


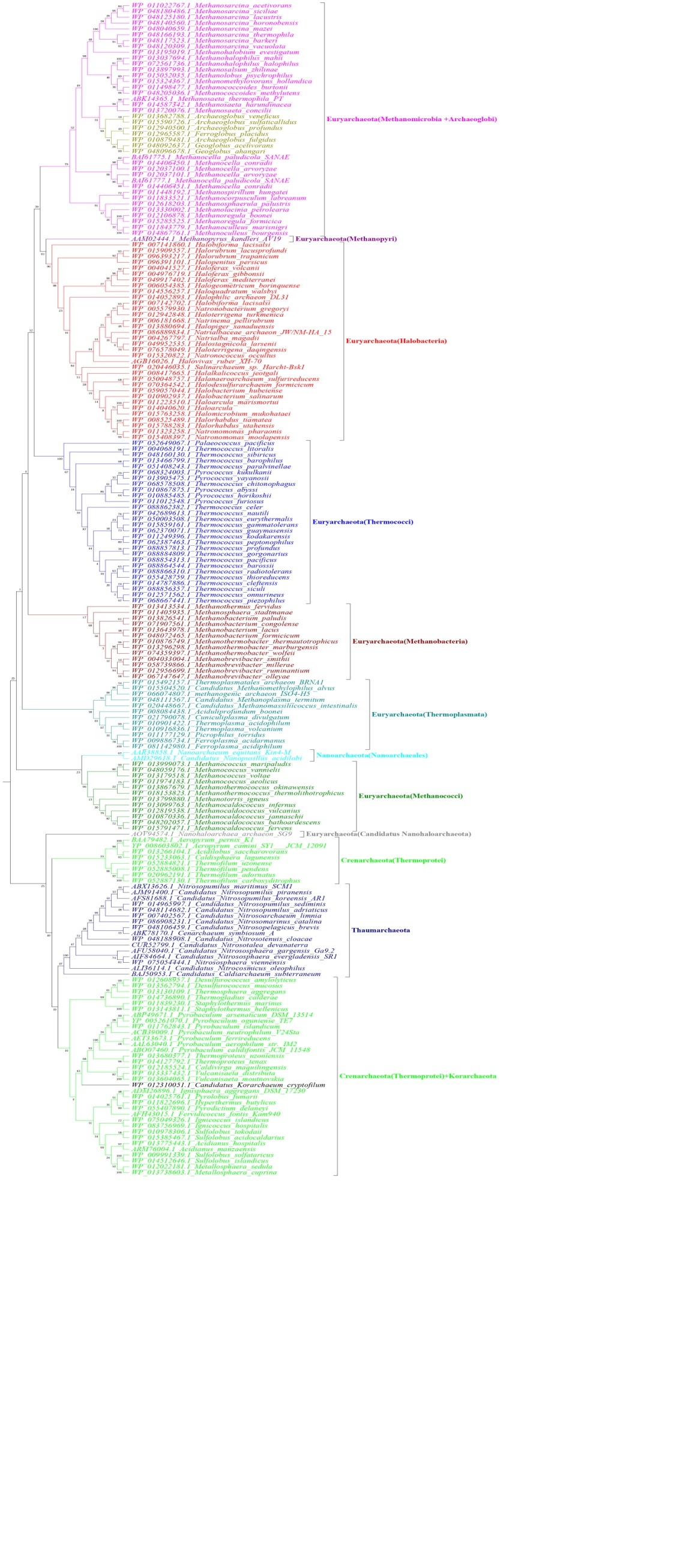


**Figure S4**: Phylogenetic tree of long-type archaeal FKBP. The tree of 201 sequences divides into a clade specific manner.


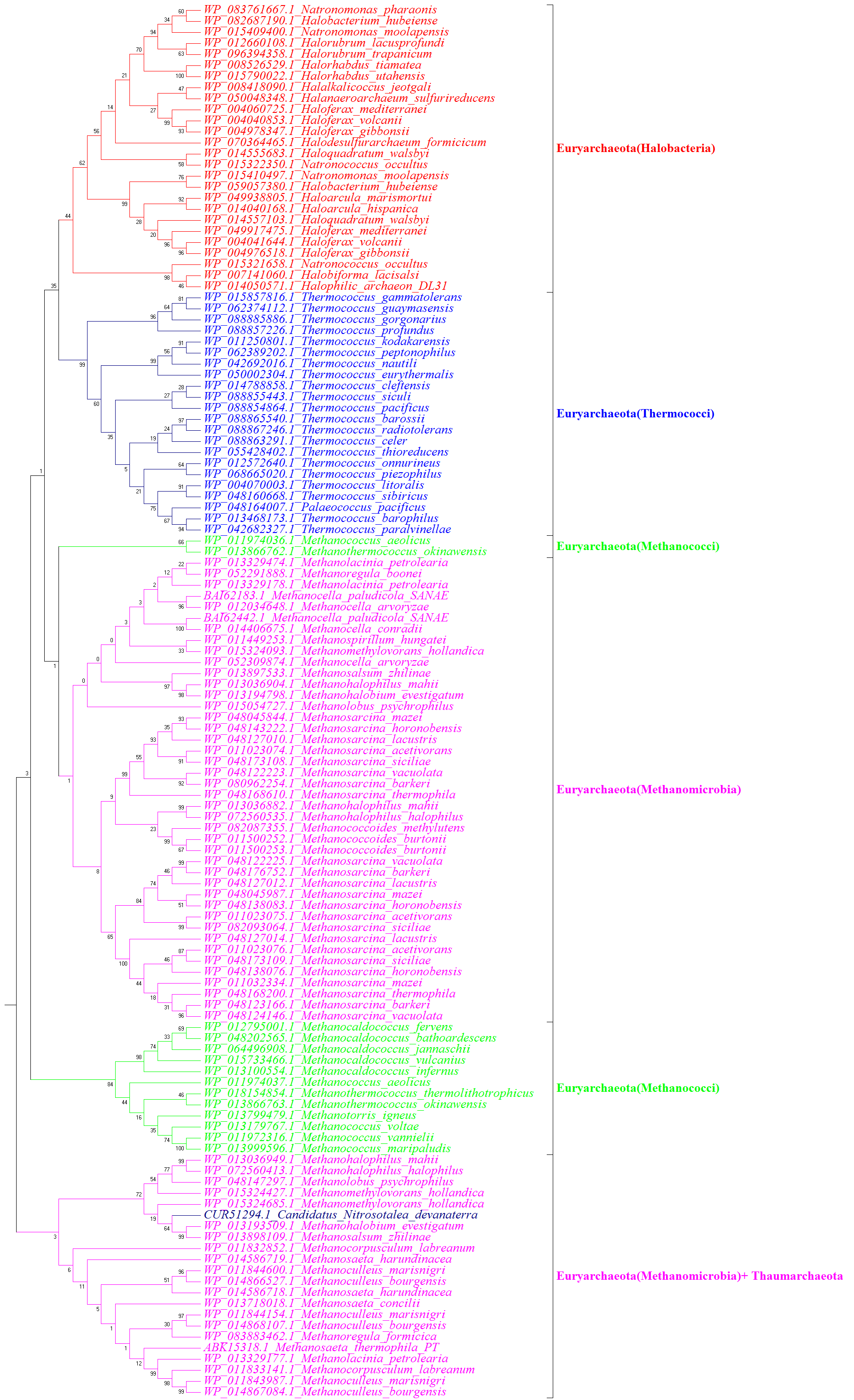


**Figure S5**: Phylogenetic tree of short-type archaeal FKBP. The tree of 126 sequences divides into different classes of Euryarchaeota in a clade specific manner.


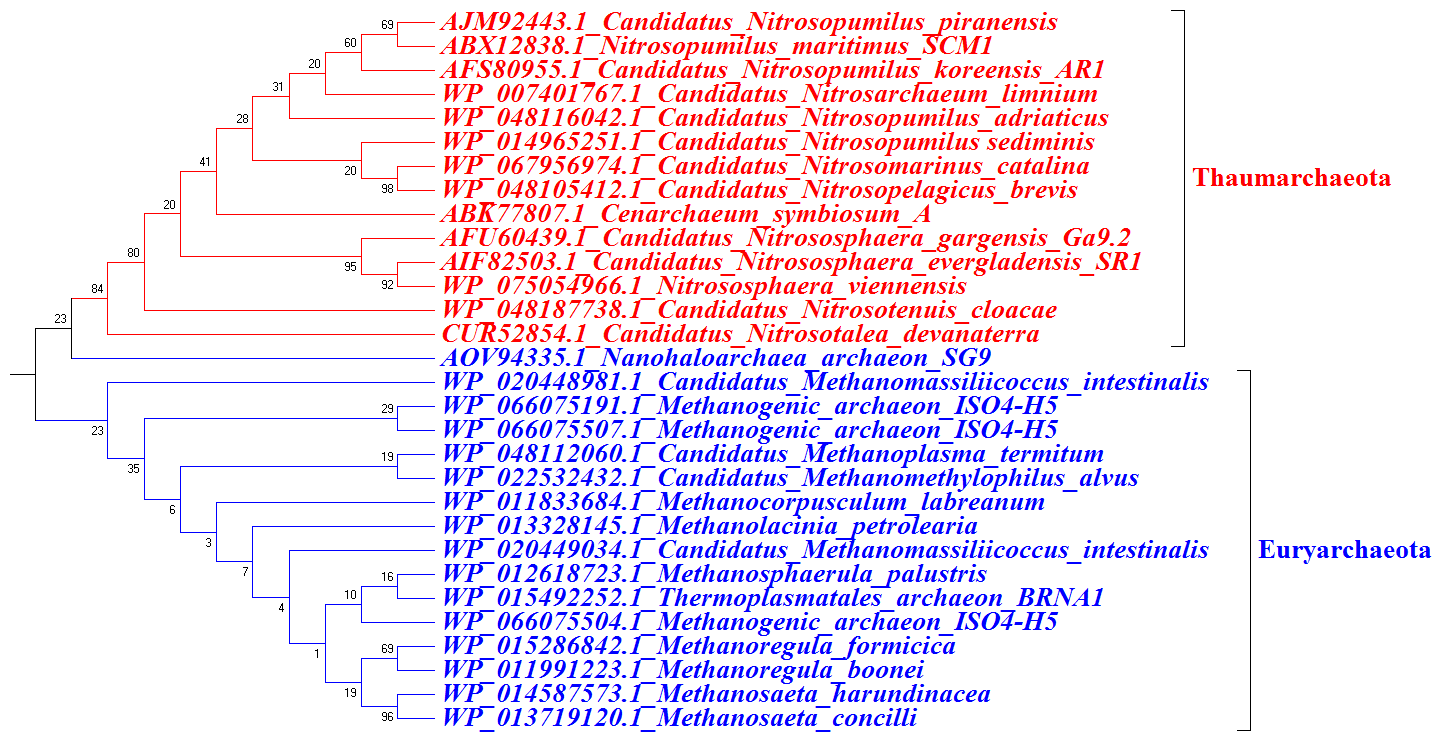


**Figure S6**: Phylogenetic tree of archaeal parvulin protein sequences. The tree of 30 sequences divides into two clades. Phylum Thaumarchaeota and Euryarchaeota highlighted in red and blue.
